# Lipid A Has Significance for Optimal Growth of *Coxiella burnetii* in Macrophage-Like THP-1 Cells and to a Lesser Extent in Axenic Media and Non-phagocytic Cells

**DOI:** 10.3389/fcimb.2018.00192

**Published:** 2018-06-08

**Authors:** Tao Wang, Yonghui Yu, Xiaofei Liang, Shengdong Luo, Zemin He, Zhihui Sun, Yongqiang Jiang, Anders Omsland, Pei Zhou, Lihua Song

**Affiliations:** ^1^State Key Laboratory of Pathogen and Biosecurity, Beijing Institute of Microbiology and Epidemiology, Beijing, China; ^2^Department of Chemistry, Duke University, Durham, NC, United States; ^3^Paul G. Allen School for Global Animal Health, College of Veterinary Medicine, Washington State University, Pullman, WA, United States; ^4^Department of Biochemistry, Duke University Medical Center, Durham, NC, United States

**Keywords:** Q fever, *Coxiella burnetii*, LpxC inhibitor, LPC-011, lipid A, LPS

## Abstract

Lipid A is an essential basal component of lipopolysaccharide of most Gram-negative bacteria. Inhibitors targeting LpxC, a conserved enzyme in lipid A biosynthesis, are antibiotic candidates against Gram-negative pathogens. Here we report the characterization of the role of lipid A in *Coxiella burnetii* growth in axenic media, monkey kidney cells (BGMK and Vero), and macrophage-like THP-1 cells by using a potent LpxC inhibitor -LPC-011. We first determined the susceptibility of *C. burnetii* LpxC to LPC-011 in a surrogate *E. coli* model. In *E. coli*, the minimum inhibitory concentration (MIC) of LPC-011 against *C. burnetii* LpxC is < 0.05 μg/mL, a value lower than the inhibitor's MIC against *E. coli* LpxC. Considering the inhibitor's problematic pharmacokinetic properties *in vivo* and *Coxiella*'s culturing time up to 7 days, the stability of LPC-011 in cell cultures was assessed. We found that regularly changing inhibitor-containing media was required for sustained inhibition of *C. burnetii* LpxC in cells. Under inhibitor treatment, *Coxiella* has reduced growth yields in axenic media and during replication in non-phagocytic cells, and has a reduced number of productive vacuoles in such cells. Inhibiting lipid A biosynthesis in *C. burnetii* by the inhibitor was shown in a phase II strain transformed with chlamydial *kdtA*. This exogenous KdtA enzyme modifies *Coxiella* lipid A with an α-Kdo-(2 → 8)-α-Kdo epitope that can be detected by anti-*chlamydia* genus antibodies. In inhibitor-treated THP-1 cells, *Coxiella* shows severe growth defects characterized by poor vacuole formation and low growth yields. *Coxiella* progenies prepared from inhibitor-treated cells retain the capability of normally infecting all tested cells in the absence of the inhibitor, which suggests a dispensable role of lipid A for infection and early vacuole development. In conclusion, our data suggest that lipid A has significance for optimal development of *Coxiella*-containing vacuoles, and for robust multiplication of *C. burnetii* in macrophage-like THP-1 cells. Unlike many bacteria, *C. burnetii* replication in axenic media and non-phagocytic cells was less dependent on normal lipid A biosynthesis.

## Introduction

*Coxiella burnetii* is a geographically widely distributed, Gram-negative intracellular bacterium. It is the causative agent of Q fever which may manifest in humans as an acute disease (mainly as a self-limiting febrile illness, pneumonia, or hepatitis) or as a chronic disease (mainly endocarditis in patients with previous valvulopathy) (Maurin and Raoult, 1999). The majority (~50–60%) of human infections are asymptomatic (Maurin and Raoult, 1999; Hechemy, 2012). Resolution of symptoms does not mean the patient is clear of infection (Harris et al., 2000). Chronic infections are rare but can be fatal if untreated. *C. burnetii* is a significant cause of culture-negative endocarditis in the United States (Mulye et al., 2017). Treatment of chronic infections is challenging and currently requires a combined antibiotic therapy with doxycycline and hydroxychloroquine for a minimum of 18 months (Angelakis and Raoult, 2010).

*C. burnetii* is the only known bacterium that replicates within acidified, degradative phagolysosome-like vacuoles (termed *Coxiella*-containing vacuole, or CCV) of eukaryotic cells. Similar to *Chlamydia* spp., it has two morphologically distinct cell types that comprise a biphasic developmental cycle (Waag, 2007). A small cell variant (SCV), likely the extracellular survival form, invades the host and develops into a large cell variant (LCV) for replication. The LCV replicates and its progenies differentiate back into SCVs during the stationary phase of the organism's growth cycle. Both the SCV and LCV forms of *Coxiella* are infectious (Wiebe et al., 1972; Minnick and Raghavan, 2012).

Gram-negative bacteria contain a principle component called lipopolysaccharide (LPS) in the outer leaflet of the outer membrane. LPS protects Gram-negative bacteria against external damaging agents such as antibiotics and detergents. It consists of a membrane saccharolipid called lipid A, a core oligosaccharide, and a distal repeating polysaccharide units (Raetz et al., 2007). Lipid A is essential for growth of most Gram-negative bacteria, and its biosynthetic pathway is an attractive target for the development of novel antibiotics (Barb and Zhou, 2008; Zhou and Zhao, 2017). Diverse inhibitors targeting LpxC, an enzyme responsible for the first committed step in lipid A biosynthesis, have been synthesized (Kalinin and Holl, 2017). These inhibitors represents a class of promising antibiotic candidates, and are new tools for studying biosynthesis and function of lipid A or LPS in Gram-negative bacteria (Nguyen et al., 2011; Tomaras et al., 2014).

Virulent *C. burnetii* harbors LPS like other Gram-negative bacteria, but undergoes an irreversible modification of its LPS, termed phase variation, when extensively passaged in immunoincompetent hosts. The phase variation is a transition of *C. burnetii* from a virulent phase I to an avirulent phase II state (Hackstadt, 1988). LPS from phase I *C. burnetii* contains two unique biomarkers of methylated sugars (virenose and dihydrohydroxystreptose) at its O-specific chain, while LPS from phase II *C. burnetii* is severely truncated and only contains lipid A and partial core oligosaccharide. LPS from phase I *C. burnetii* may mask toll-like receptor ligands from innate immune recognition by human dendritic cells, thus might play an important role in *C. burnetii* persistent infections (Shannon et al., 2005). Before the advent of axenic culture (Omsland et al., 2009) and modern genetic techniques in *C. burnetii* (Beare and Heinzen, 2014), the investigation of biology and pathogenesis of *C. burnetii* LPS mutants was limited to the use of naturally occurring LPS mutants (Narasaki and Toman, 2012; Larson et al., 2016). Thus far LPS is the only *C. burnetii* virulence factor evaluated in immunocompetent rodent models (mice and guinea pigs) (Moos and Hackstadt, 1987; Andoh et al., 2007).

Lipid A is the basal component of LPS, and is termed endotoxin due to its role in LPS toxicity. The lipid A moiety plus one 3-deoxy-D-*manno*-oct-2-ulosonic acid (Kdo) is the minimal requirement for survival of many Gram-negative bacteria. In *Chlamydia trachomatis*, also an intracellular vacuole pathogen, lipid A is essential for the generation of infectious progeny (Nguyen et al., 2011). *C. burnetii* lipid A is well characterized (Narasaki and Toman, 2012). Its proximal region contains three Kdo residues–one of which is suggested to be a Kdo-like substance, likely a Ko (modified from Kdo by a KdoO enzyme) (Toman and Skultety, 1994; Chung and Raetz, 2011; Narasaki and Toman, 2012). Endotoxin activities of *C. burnetii* lipid A were up to 1,000-fold less active than lipid A of *E. coli* (Amano et al., 1987). Though various biological and immunomodulatory functions of *C. burnetii* LPS have been described, the importance of lipid A in *C. burnetii* biology remains unclear.

In this study, we characterized the role of lipid A in *C. burnetii* growth in different culture systems by using a potent LpxC inhibitor -LPC-011. We first evaluated the minimum inhibitory concentration (MIC) of LPC-011 against *C. burnetii* LpxC in a surrogate *E. coli* model. A reliable condition of effectively inhibiting lipid A biosynthesis in *C. burnetii* was then established in a transformant carrying *Chlamydia trachomatis kdtA*. Inhibitory effects of LPC-011 against *C. burnetii* were tested with phase I and phase II strains. Our data suggest that lipid A partially contributes to the formation of productive *Coxiella*-containing vacuoles in non-phagocytic cells, and has significance for *C. burnetii* robust multiplication in macrophage-like THP-1 cells, but to a much lesser extent in axenic media and non-phagocytic cells. Our data suggest that LpxC inhibitors have potential as anti-*C. burnetii* agents during infection.

## Materials and methods

### Cell lines, bacterial strains, and key reagents

African monkey kidney cells (Vero) and Buffalo green monkey kidney cells (BGMK) were grown in high-glucose-containing Dulbecco's Modified Eagle Medium (DMEM) supplemented with 10% FBS. Human monocytic leukemia cells (THP-1) were cultured in RPMI 1640 medium supplemented with 10% FBS. *C. burnetii* Nine Mile phase II, Henzerling phase I and *Chlamydia trachomatis* B/QH111L are from our laboratory strain collection. All media and FBS were purchased from Life Technologies (Beijing, China). Synthesis of the LpxC inhibitor, LPC-011, was described previously (Liang et al., 2011). PCR primers used in this study are shown in Table S1.

### Construction of an *E. coli* mutant carrying *C. burnetii lpxC* with an intron insertion in the chromosomal copy of *lpxC*

A DNA fragment including sequences flanking *C. burnetii lpxC* (CBU_0142) was amplified from *C. burnetii* Nine Mile phase II genomic DNA with primers CBlpxCF and CBlpxCR, and was cloned into a T vector of pZeroBack/Blunt (TIANGEN Biotech, Beijing, China). The resulting plasmid pCBlpxC was transformed into *E. coli* BL21(DE3). The chromosomal copy of *E. coli lpxC* was disrupted by insertion of a group II intron using a commercial TargeTron gene knockout system (TA0100, Sigma-Aldrich). The target site for TargeTron insertion was between nucleotide residues 326 and 327 on the sense strand. Four PCR primers –IBS1/2, EBS1/delta, EBS2, and EBS/Universal, were used to modify the intron for insertion into the predicted site according to the TargeTron system user guide (sigma-aldrich.com/targetron).

### Construction of a shuttle vector for lipid a-modification in *C. burnetii*

The *C. burnetii* shuttle vector pMMGKkdtA was constructed by cloning kanamycin resistance (Kan^R^) cassette, eGFP and *C. trachomatis kdtA* gene into RSF1010 *ori*-based vector pMMB207, kindly provided by Xuehong Zhang (Shanghai Jiaotong University, Shanghai, China). The Kan^R^, eGFP and *kdtA* genes were driven by *C. burnetii* promoters of CBU1169, CBU0311 and CBU1169, respectively. Promoter regions of CBU0311 and CBU1169 were amplified with P311-Kpn1F/P311-R and P1169-F/P1169-R, respectively. The Kan^R^ and eGFP genes were amplified from pEASY-T1 and pEGFP-C1, with Kan-F/Kan-XhoI-R and eGFP-F/eGFP-R, respectively. Overlapping PCR with primers P311-KpnI-F/Kan-XhoI-R was used to produce the P311-eGFP-P1169-Kan fragment. The CBU1169 promoter region and the *C. trachomatis kdtA* gene were amplified with P1169-NheI-F/P1169-kdtA-R and kdtA-NheI-F/kdtA-XhoI-R, respectively, and then overlapping PCR with primers P1169-NheI-F/kdtA-XhoI-R to generate the P1169-kdtA segment. The kdtA-P1169-P311-eGFP-P1169-Kan fragment was then amplified with primers kdtA-XhoI-R/Kan-KpnI-R. The RSF1010 *ori*-contained fragment was amplified from pMMB207 with p207-XhoI-F/p207-KpnI-R. The two fragments were digested with *Kpn*I and *Xho*I, and ligated to generate the vector pMMGKkdtA (Datasheet S1). The resulting vector was transformed into *C. burnetii* Nine Mile phase II by electroporation (Beare and Heinzen, 2014). *C. burnetii* transformants were cloned three successive times in ACCM-2 plates and were expanded in BGMK cells (Beare and Heinzen, 2014).

### *C. burnetii* culture and purification

All *C. burnetii* strains (Nine Mile phase II, Henzerling phase I and transformants of Nine Mile phase II) were purified from BGMK cultures by centrifugation through a 30% Renografin density gradient. Their progenies generated under LpxC inhibitor treatment were purified from BGMK cultures with regularly changed cell media containing 2 μg/mL LPC-011 every 48 h. Purified organisms were suspended in SPG (0.25 M sucrose, 10 mM sodium phosphate, 5 mM L-glutamic acid), and kept frozen at −80°C. For testing the inhibitory effects of LPC-011 in axenic culture, ACCM-2 medium plus 500 μM tryptophan was used (Vallejo Esquerra et al., 2017).

### Activity assessment of LPC-011 in spent media

Spent media in cell culture or axenic culture of *C. burnetii* were collected at indicated time points, then were centrifuged at 20,000 g for 10 min to remove bacteria or cell debris and kept frozen at −80°C. To assess the activity of LPC-011, *E. coli* TOP10 was added into 1 mL spent media in 1.5 mL tubes and cultured overnight at 37°C with shaking (220 rpm). The optical density at 550 nm (OD_550_) was measured. All samples were performed in triplicate.

### *C. burnetii* infections and phase contrast microscopy

Monolayers of cells (Vero, BGMK, or THP-1) were grown in appropriate media as described above in 24-well plates and were infected with parent *C. burnetii* strains or their progenies generated under LPC-011 treatment at appropriate MOIs. Before infecting THP-1 cells, cultures were treated with 200 nM phorbol 12-myristate 13-acetate (PMA) for 24 h for differentiation into macrophage-like cells as previously described (Larson et al., 2015). To facilitate infection, plates were centrifuged at 800 g for 30 min, then was washed with PBS and 1 ml RPMI 1640 medium supplemented with 5% FBS was added per well. Medium not supplemented with FBS was used for the duration of BGMK infections. All infections were performed in triplicate. Plates were incubated at 37°C. Phase-contrast images were captured at a magnification of × 400 by using a Nikon eclipse TS100 inverted microscope to record the vacuole morphology of *C. burnetii* strains.

### Immunofluorescence

Cells grown on coverslips were infected with *C. burnetii* as described above. At 7 days post-infection, cells were fixed with ice-cold methanol for 10 min at room temperature. After washing with PBS, cells were subjected to antibody and chemical staining. Human sera from chronic Q fever patients plus an anti-human IgG conjugated with Alexa Fluor 488 were used to visualize *C. burnetii*. Lipid A of *C. burnetii* transformants carrying *C. trachomatis kdtA* was stained with mouse anti-*Chlamydia* genus monoclonal antibodies (Santa Cruz Biotech) and a rabbit anti-mouse IgG-PE conjugate (Life Technologies). An Olympus FV1000S laser confocal microscope (Olympus) was used to analyze the stained samples.

### Quantitative PCR

Purified *C. burnetii* recovered from host cell and axenic cultures were quantified by using qPCR as previously described with minor modifications (Coleman et al., 2004). Cell samples were harvested by using trypsin (Hyclone) treatment and centrifugation. Total DNA was extracted with DNeasy Blood Tissue Kit (Qiagen). Genome copy numbers were determined by Taqman probe qPCR specific to *dotA* by using an ABI 7300 sequence detection system (Applied Biosystems). PCR conditions were as follows: initial denaturation at 94°C for 10 min, followed by 40 cycles of amplification at 94°C for 15 s, 60°C for 1 min.

### Transmission electron microscopy

Cell were fixed with 0.1 M phosphates/2.5% glutaraldehyde buffer (pH = 7.4) for 4 h, then incubated in 1% cacodylate-buffered osmic acid and treated with 1% uranyl acetate in distilled water. Samples were dehydrated with graded amounts of acetone and embedded in Spurr's resin. Ultrathin sections were stained with 1% uranyl acetate and Reynold's lead citrate. Images were captured with a Hitachi H-7600 transmission electron microscope (Hitachi).

### Statistical analysis

A two-tailed Student *t* test was used for qPCR analysis of *C. burnetii* growth yields under various conditions. The standard deviation was determined from three independent biological replicates.

## Results

### A single high dose of LpxC inhibitors cannot restrict *C. burnetii* growth in vero cells

**Figure 1:**
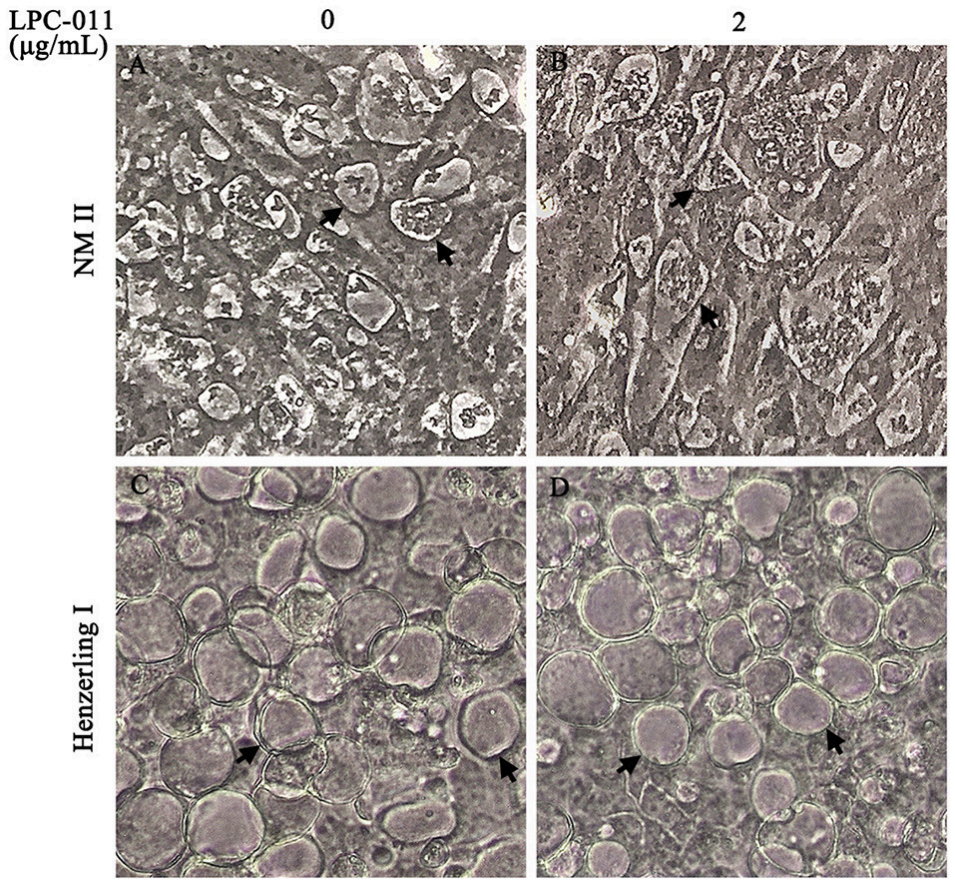
*C. burnetii* CCVs appear normal on Vero cells with a single dose (2 μg/mL) of LPC-011. Monolayers of *C. burnetii* Nine Mile phase II **(A,B)** and Henzerling phase I **(C,D)** infected cells with or without adding LPC-011 were imaged by phase microscopy at 7 days post-infection. Arrows indicate CCVs.

Various cell lines have been used for culture and antibiotic susceptibility testing of *C. burnetii* (Angelakis and Raoult, 2010). When extensively passaged in cell cultures, full-length LPS of phase I *C. burnetii* tends to become severely truncated while lipid A remains unaffected, suggesting selective pressure helps to retain the lipid A component of LPS. Because lipid A is essential for the viability of most Gram-negative bacteria, we tested the effectiveness of a broad-spectrum LpxC inhibitor –LPC-011 as a potential anti-*Coxiella* agent. In preliminary experiments, Vero cells were infected using a high MOI (>50) of phase I and phase II *C. burnetii* strains by centrifugation at 800 × g for 30 min, and fresh media containing different doses (0, 1–10 μg/mL) of LPC-011 were added after infection. Vacuole formation and bacterial growth were assessed by phase contrast microscopy for over 7 days. Unexpectedly, no perceptible phenotypes were observed during cell cultures of *C. burnetii*, although similar cell toxicity was observed at high concentrations of LPC-011 (Nguyen et al., 2011), suggesting lipid A could be dispensable for *C. burnetii* culture in Vero cells. The *C. burnetii* culture in the presence or absence of 2 μg/mL LPC-011 is shown in Figure 1. However, a conclusive role of the essentiality of lipid A in *C. burnetii* growth cannot be determined due to the lack of a method to detect the disruption of lipid A biosynthesis. To measure the potential MIC of LPC-011 against *C. burnetii* during intracellular replication is further complicated by the pathogen's lengthy developmental cycle inside acidic vacuoles, and the known difference of MICs of LPC-011 for intracellular vs. extracellular growth of the same bacterium such as *Salmonella typhimurium* (Nguyen et al., 2011).

### LPC-011 inhibits *Coxiella* LpxC at 0.05 μg/mL in a surrogate *E. coli* system

**Figure 2:**
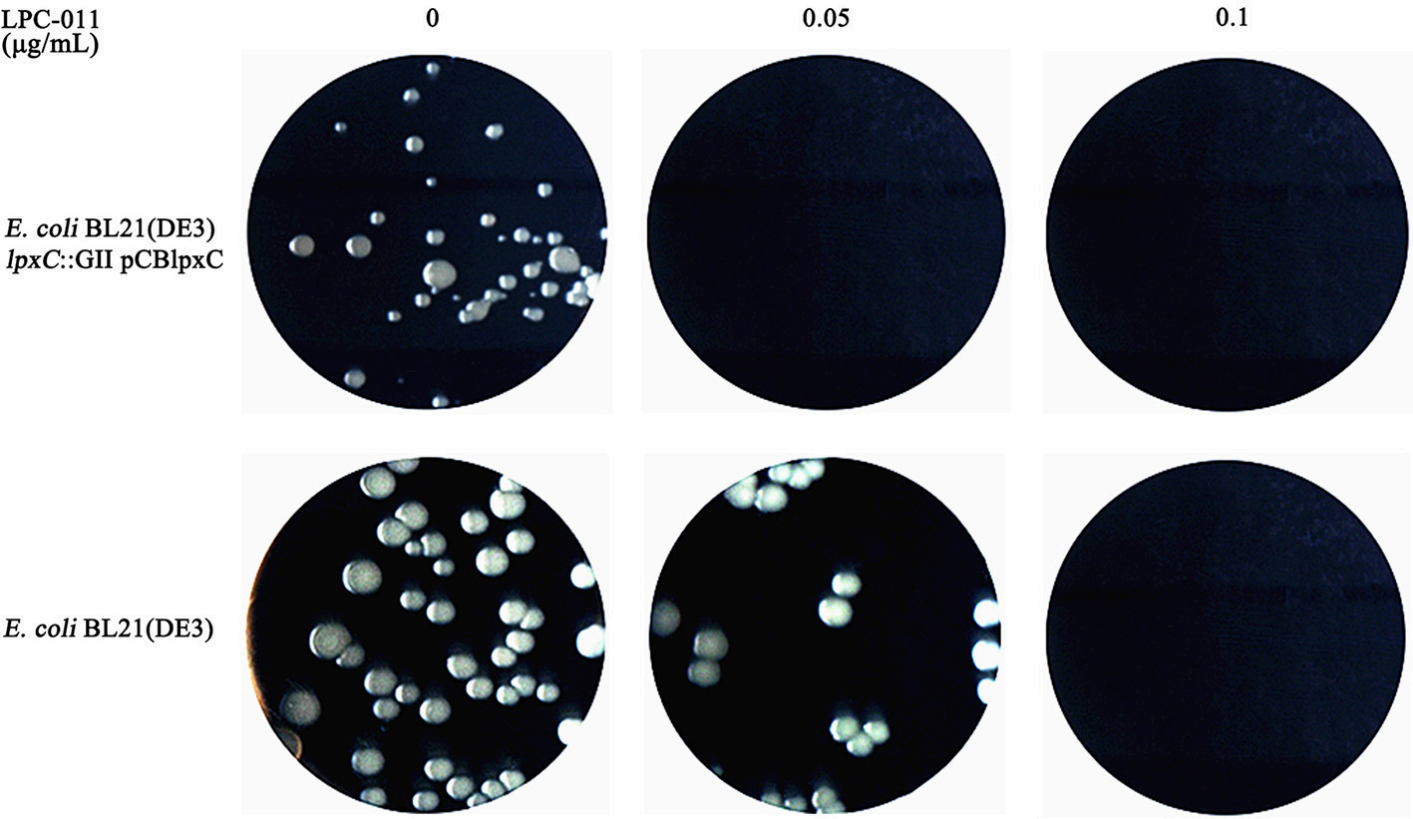
Growth comparison of wild type *E. coli* and an *E. coli* mutant carrying *C. burnetii lpxC* on LB-agar plates containing different doses of LPC-011. The *E. coli* mutant (*E. coli* BL21(DE3) *lpxC*::GII pCBlpxC) was constructed by inserting a group II intron in the chromosomal *lpxC* gene of an *E. coli* transformant carrying the pCBlpxC plasmid, which contains *C. burnetii lpxC*.

LPC-011 is reportedly a broad-spectrum inhibitor of LPS biosynthesis against a variety of Gram-negative pathogens. Indeed, the MIC of LPC-011 against *E. coli* W3110 is as low as 0.03 μg/mL (Liang et al., 2011). We next sought to determine the susceptibility of *C. burnetii* LpxC to LPC-011 in a surrogate *E. coli* model (Figure 2), as it has been shown that susceptibility of *E. coli* to LpxC inhibitors is dependent on the source of *lpxC* gene (Lee et al., 2011; Liang et al., 2011). The *C. burnetii* LpxC shares 55% amino acid identity with the *E. coli* LpxC, including eight identical amino acids at the active site (Coggins et al., 2003; Whittington et al., 2003). *E. coli* BL21(DE3) was transformed with a plasmid carrying *C. burnetii lpxC*, and the chromosomal *lpxC* was inactivated by inserting a group II intron. The successful disruption of *E. coli lpxC* suggests that *C. burnetii lpxC* can complement the essential function of the endogenous *E. coli lpxC* in *trans*. In LB plates containing 0.05 μg/mL of LPC-011, the *E. coli* mutant complemented with *C. burnetii lpxC* failed to grow while growth of the wild type *E. coli* was not affected, suggesting LPC-011 has an MIC against *C. burnetii* LpxC below 0.05 μg/mL. Importantly this MIC is lower than the MIC against *E. coli* LpxC –between 0.05 and 0.1 μg/mL. Compared to the previous reported MIC (0.03 μg/mL) of LPC-011 against *E. coli* W3110 using liquid cultures (Liang et al., 2011), our measured MIC value of LPC-011 against *E. coli* BL21(DE3) is slightly higher, which may reflect the difference of assaying methods (liquid culture vs. plating).

### Reduced growth of *C. burnetii* in axenic medium containing 5 μg/mL LPC-011

**Figure 3:**
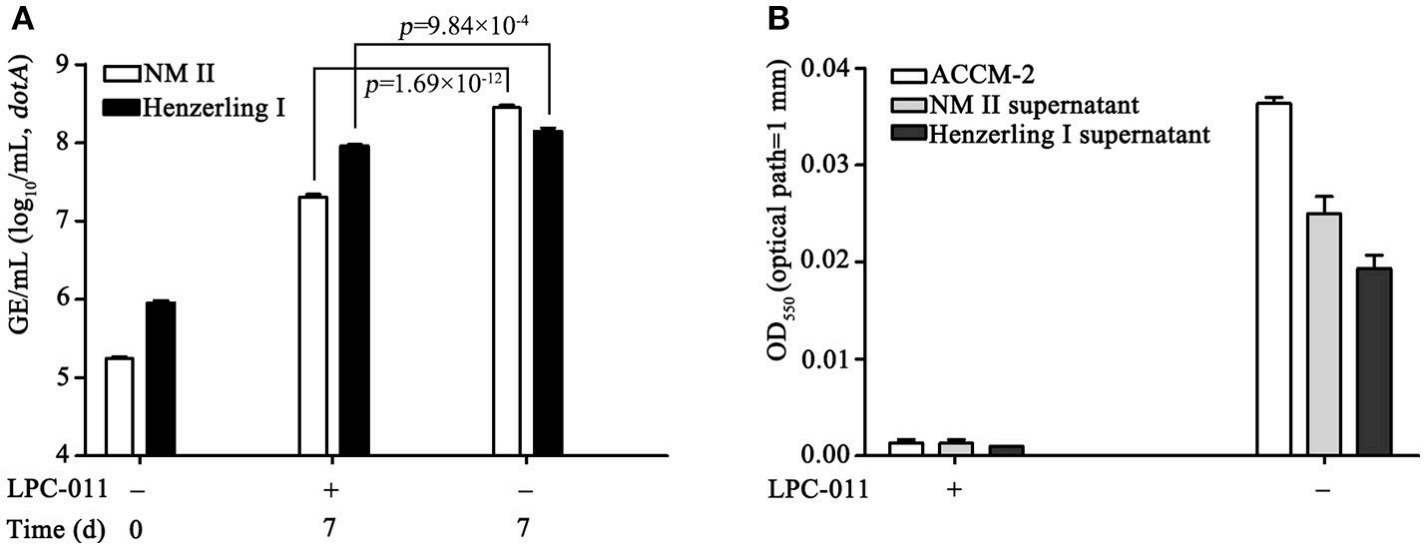
Analysis of *C. burnetii* growth in axenic media containing a constant *E. coli*-lethal concentration of LPC-011. **(A)** Growth of *C. burnetii* Nine Mile phase II and Henzerling phase I in axenic media with or without 5 μg/mL LPC-011 were quantified by qPCR. **(B)** Overnight growth of *E. coli* TOP10 in axenic media with or without 5 μg/mL LPC-011 after *C. burnetii* inoculation for 7 days.

ACCM-2 supplemented with tryptophan is an acidified citrate cysteine medium that was recently modified for improved culture of *C. burnetii* (Omsland et al., 2009, 2011; Vallejo Esquerra et al., 2017). It can support 3–4 logs of growth of *C. burnetii* after 7 days of cultivation in 5% O_2_ at 37°C. We tested if a single dose (5 μg/mL, >100 × MIC) of LPC-011 affects *C. burnetii* growth in ACCM-2 plus tryptophan. Under control conditions, *C. burnetii* phase I and phase II strains normally grew 2.2 and 3.2 logs, compared to 2.0 and 2.1 logs of increase with LPC-011 treatment, respectively (Figure 3A, Table S2). Between the two strains, the growth of the phase II strain was more affected by the inhibitor (Table S2). The growth of *E. coli* in spent ACCM-2 was used to assess the activity of LPC-011 (Figure 3B). The spent ACCM-2 with LPC-011 maintained an *E. coli*-lethal activity, while the spent ACCM-2 without LPC-011 appeared to affect *E. coli* growth differentially. In the absence of LPC-011, compared to the phase II strain, the phase I strain had lower growth yields and unexpectedly its spent media supported lower growth yields of *E. coli*, which might reflect the differential nutrient consumption by different *C. burnetii* strains. As the same inhibitor concentration in *C. burnetii* growth media maintains an *E. coli*-lethal effect during the 7-day incubation period and as *C. burnetii* LpxC is more susceptible to LPC-011 than *E. coli* LpxC (Figure 2), it is reasonable to conclude that the reduced growth phenotype of *C. burnetii* arises from LpxC inhibition and that lipid A biosynthesis is not essential for *C. burnetii* growth in axenic media.

### Regular refreshment of LPC-011 is required for consistent inhibition of *C. burnetii* LpxC in cell cultures

**Figure 4:**
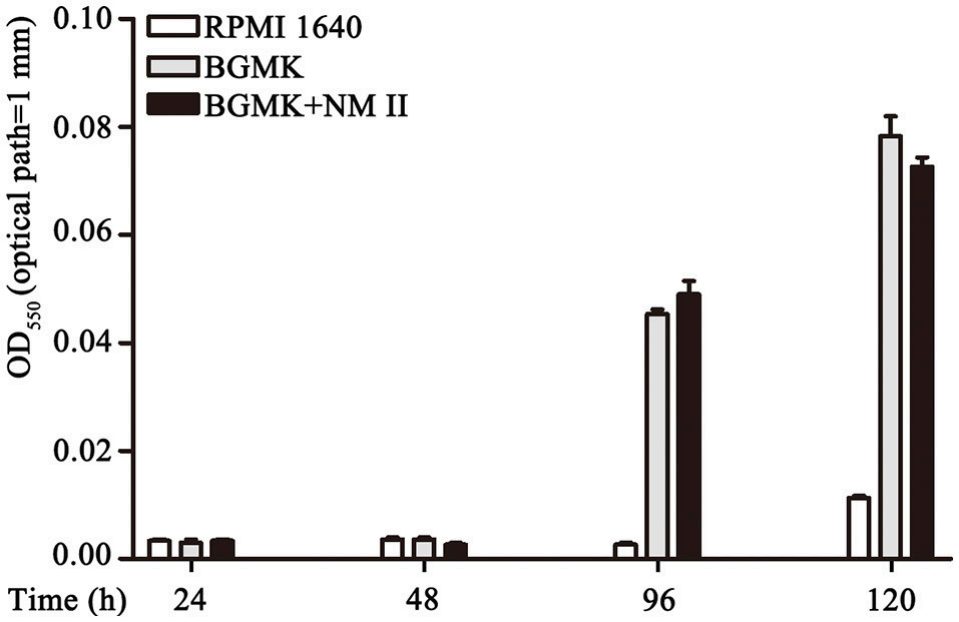
Stability assessment of LPC-011 in *C. burnetii* cell culture. Overnight growth of *E. coli* in LPC-011-containing (2 μg/mL) cell media collected at different time points after incubation at 37°C, 5% CO_2_ was measured. Three groups—media alone, normal cell culture, and *C. burnetii* infected cells were included.

LPC-011 is a hydroxamic acid derivative. Compounds of the hydroxamate series were found to possess short pharmacokinetic half-lives when tested *in vivo* (Brown et al., 2012; Kalinin and Holl, 2017). Though LPC-011 has been tested for antibacterial activity in another obligate intracellular bacterium –*Chlamydia* (Nguyen et al., 2011; Cram et al., 2017), there is little information on the stability of LPC-011 in cell cultures. The chemical stability of LPC-011 is important in the context of the unique biology of *C. burnetii*-a 7-day growth cycle in acidic phagolysosome-like vacuoles. Therefore, we evaluated the stability of LPC-011 in BGMK cell cultures by using growth of *E. coli* in cell culture medium as an indicator (Figure 4). BGMK cells can maintain a confluent monolayer without using FBS and were used in most of our *C. burnetii* infections. By collecting media every 24 h from mock or *C. burnetii*-infected cells and culturing *E. coli* TOP10 overnight, we found that a starting concentration of 2 μg/mL LPC-011 retained an *E. coli*-inhibiting concentration for at least 48 h. Nonetheless, our data suggest that gradual depletion of the inhibitor was very likely due to the activity of BGMK cells, not *C. burnetii*. Thus, when using 2 μg/mL LPC-011 for testing its effectiveness of an anti-*C. burnetii* agent, changing media containing freshly added inhibitors every 48 h was required to maintain an *E. coli*-inhibiting concentration throughout the bacterium's growth cycle.

### LPC-011 inhibits lipid a biosynthesis in a *C. burnetii* phase II strain carrying *C. trachomatis kdtA*

**Figure 5:**
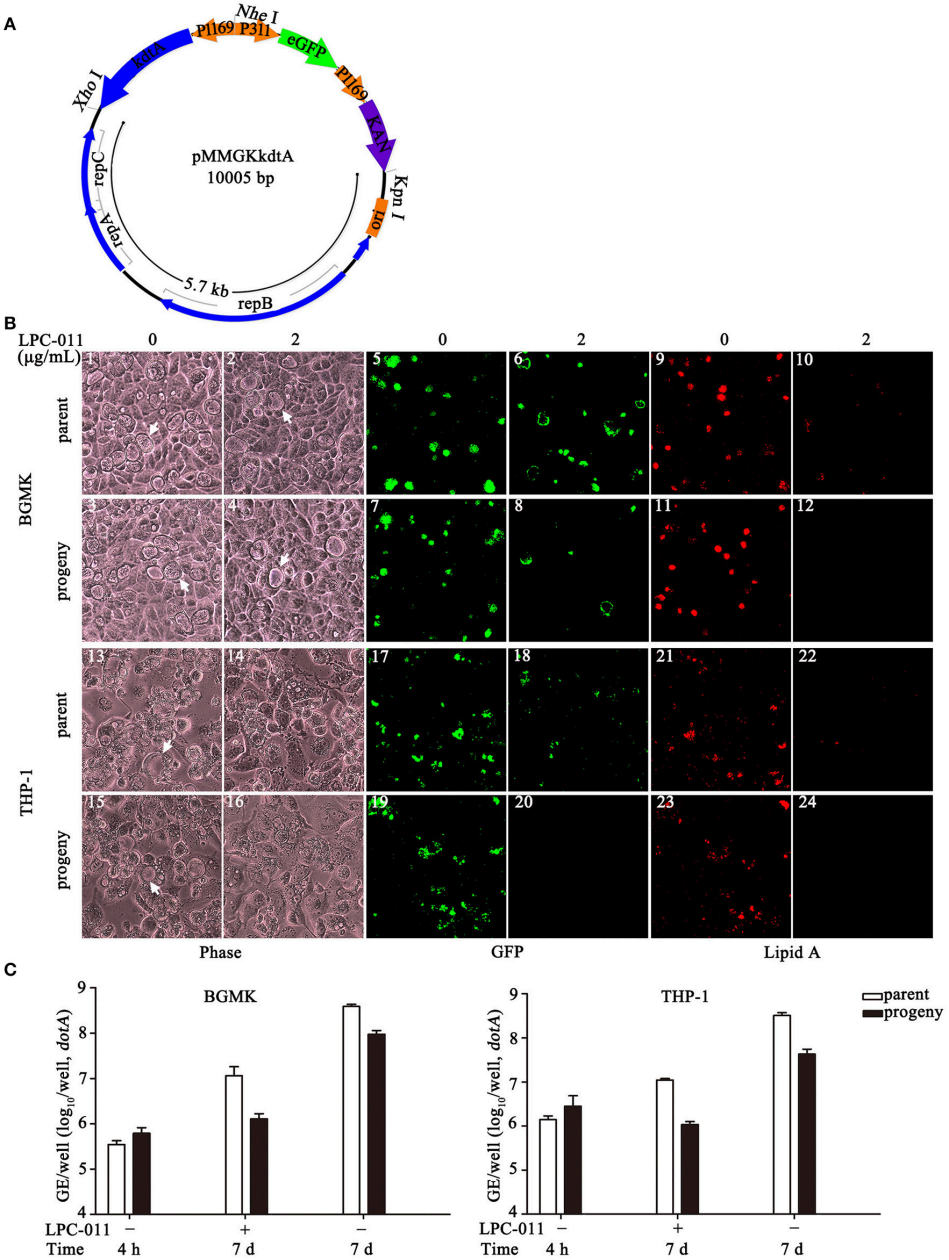
LPC-011 inhibits lipid A biosynthesis in *Cb:Ct kdtA* (a *C. burnetii* transformant carrying *C. trachomatis kdtA*). **(A)** Plasmid map of pMMGKkdtA. It has a 5.7 kb segment from pMMB207, chlamydial *kdtA* and two marker genes (eGFP and Kan^R^). **(B)** Growth of *C. burnetii* (parent *Cb:Ct kdtA* and its progeny prepared from LPC-011 treated BGMK cells) on BGMK (1–12) and THP-1 (13–24) cells with or without LPC-011 treatment. Vacuole and bacterial growth, and lipid A expression was assessed by phase microscopy, observing eGFP expression, and immunofluorescence, respectively, after 7 d growth. Arrows indicate CCVs. **(C)** Quantitative analysis of *C. burnetii* (parent *Cb:Ct kdtA* and its progeny prepared from LPC-011 treated BGMK cells) growth on BGMK and THP-1 cells with or without LPC-011 treatment.

With the above established procedures of changing media containing 2 μg/mL LPC-011 every 48 h, we investigated *C. burnetii* intracellular replication under conditions of LpxC inhibition. Whether lipid A biosynthesis of *C. burnetii* can be inhibited by LPC-011 under these conditions is unknown. We therefore established a lipid A reporter system to define the inhibition of lipid A biosynthesis in *C. burnetii* during infection of cultured cells. The *C. burnetii* phase II strain was transformed with a RSF1010 *ori*-based vector pMMGKkdtA that carries the *kdtA* gene of *Chlamydia trachomatis* (Figure 5A). The chlamydial KdtA is a tri-functional Kdo transferase (Belunis et al., 1995) and can modify *C. burnetii* lipid A with a unique α-Kdo-(2 → 8)-α-Kdo disaccharide, which can be detected by anti-*Chlamydia* genus-specific monoclonal antibodies (Figure 5B). The *C. burnetii* transformant (*C. burnetii* NM IIpMMGKkdtA, termed *Cb:Ct kdtA*) grew normally in BGMK cells. Its lipid A can be efficiently detected by immunofluorescence with anti-*Chlamydia* genus antibodies, indicating modification of lipid A as predicted (Figure 5B).

Under conditions of established LpxC inhibition in BGMK cells, the parent *Cb:Ct kdtA* strain can form normal parasitophorous vacuoles though most vacuoles do not appear to be fully filled with progeny particles after 7 days of growth and growth yield was only about 0.5 log (Figure 5C); importantly however, little or no lipid A could be detected by immunofluorescence with anti-*Chlamydia* antibodies. The *Cb:Ct kdtA* strain was expanded in BGMK cultures under LpxC inhibition, then used to infect new cultures of BGMK and THP-1 cells. These progenies grew normally in BGMK cells without using inhibitors, but had fewer vacuoles and very limited growth yields in the presence of LPC-011. In macrophage-like THP-1 cells under similar conditions of LpxC inhibition, the parent *Cb:Ct kdtA* strain had slow growth and vacuole expansion (data not shown), limited growth yield, and no lipid A could be detected. Moreover, the progenies prepared from BGMK cultures completely failed to form vacuoles upon re-infection in THP-1. However, in the absence of LPC-011, the same progenies were able to establish infection and grow normally in THP-1, suggesting lipid A is not involved in early vacuole development. Collectively, LPC-011 can effectively inhibit lipid A biosynthesis in the *Cb:Ct kdtA* strain during infection of BGMK and THP-1 cells.

### Growth of wild-type *C. burnetii* phase I and phase II on BGMK cells under constant LpxC inhibition

**Figure 6:**
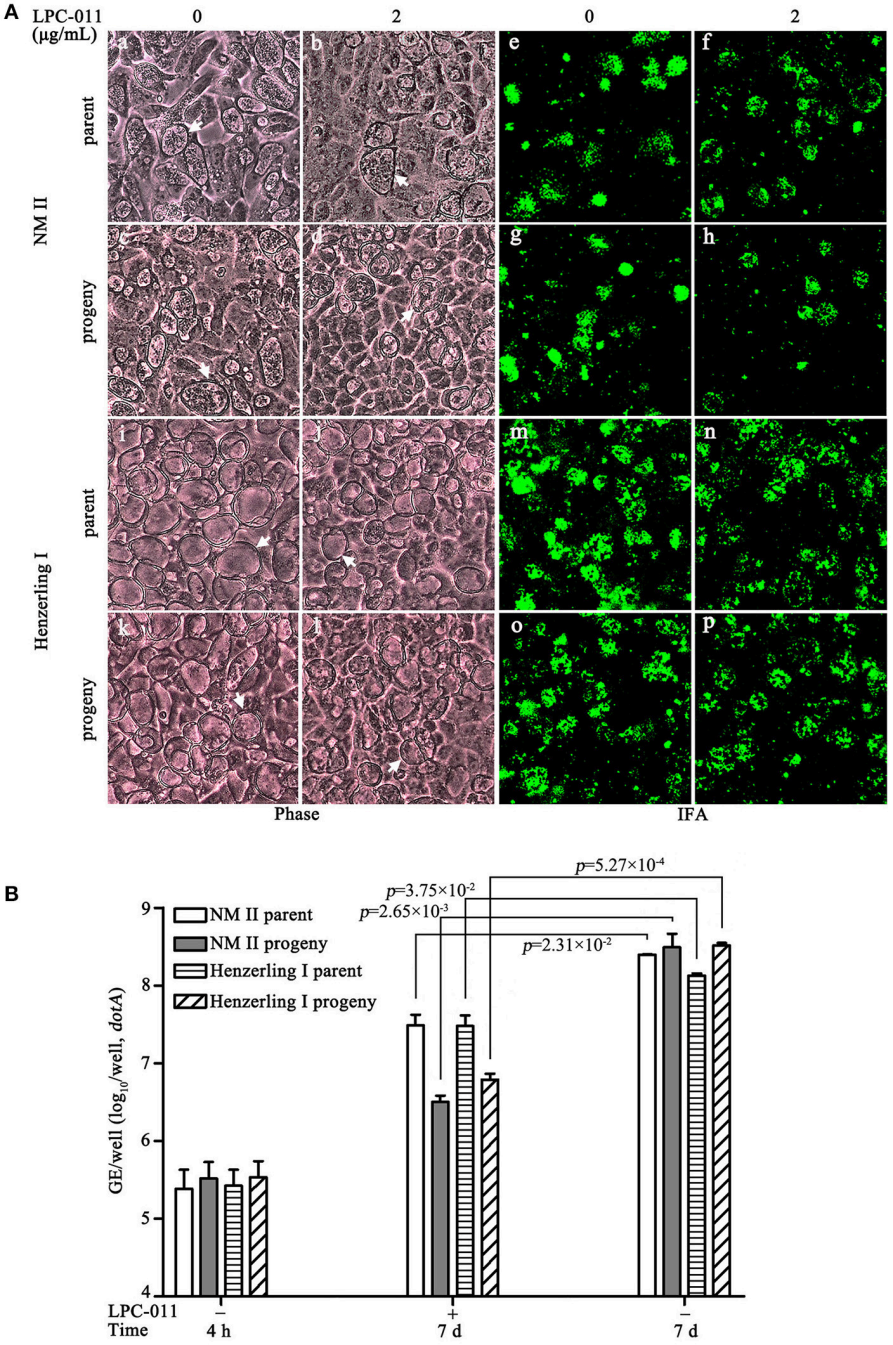
Growth of wild type *C. burnetii* in inhibitor-treated BGMK cells. **(A)** *C. burnetii* growth in BGMK cells with or without inhibitor treatment was assessed by phase contrast microcopy and immunofluorescence. Two *C. burnetii* strains –Nine Mile phase II and Henzerling phase I and their progenies prepared from inhibitor-treated BGMK cells were included. Arrows indicate CCVs. **(B)** Growth yield analysis of the above two *C. burnetii* strains in BGMK cells with or without LPC-011.

Having confirmed the inhibition of lipid A biosynthesis in cell cultures, we next compared the inhibitory effects of LPC-011 between wild type phase I and phase II strains. Similar to results of our preliminary experiments, under conditions of LpxC inhibition in BGMK cells after 7 days post-infection, both phase I and phase II strains and their progenies prepared from inhibitor-treated BGMK cells appeared to form vacuoles mostly compacted with bacterial particles (Figure 6A), in contrast to the partially filled vacuoles of the *Cb:Ct kdtA* strain. However, the inhibitor does affect growth yields of both parent strains and their progenies (*p* < 0.05) (Figure 6B, Table S2), which is at least in part due to their reduced vacuole numbers (Figure S2). The reduced number of productive vacuoles due to the inhibitor is particularly significant in low MOI infections, e.g. about 50% less vacuoles were formed in MOI = 1 infections of both strains (Figure S1). To be noted, the active Brownian movement of evenly distributed bacterial particles of the phase I strain inside the vacuoles was not affected by LPC-011, suggesting that LPS is not the only factor contributing to high hydrophilicity of the phase I strain (data not shown).

**Figure 7:**
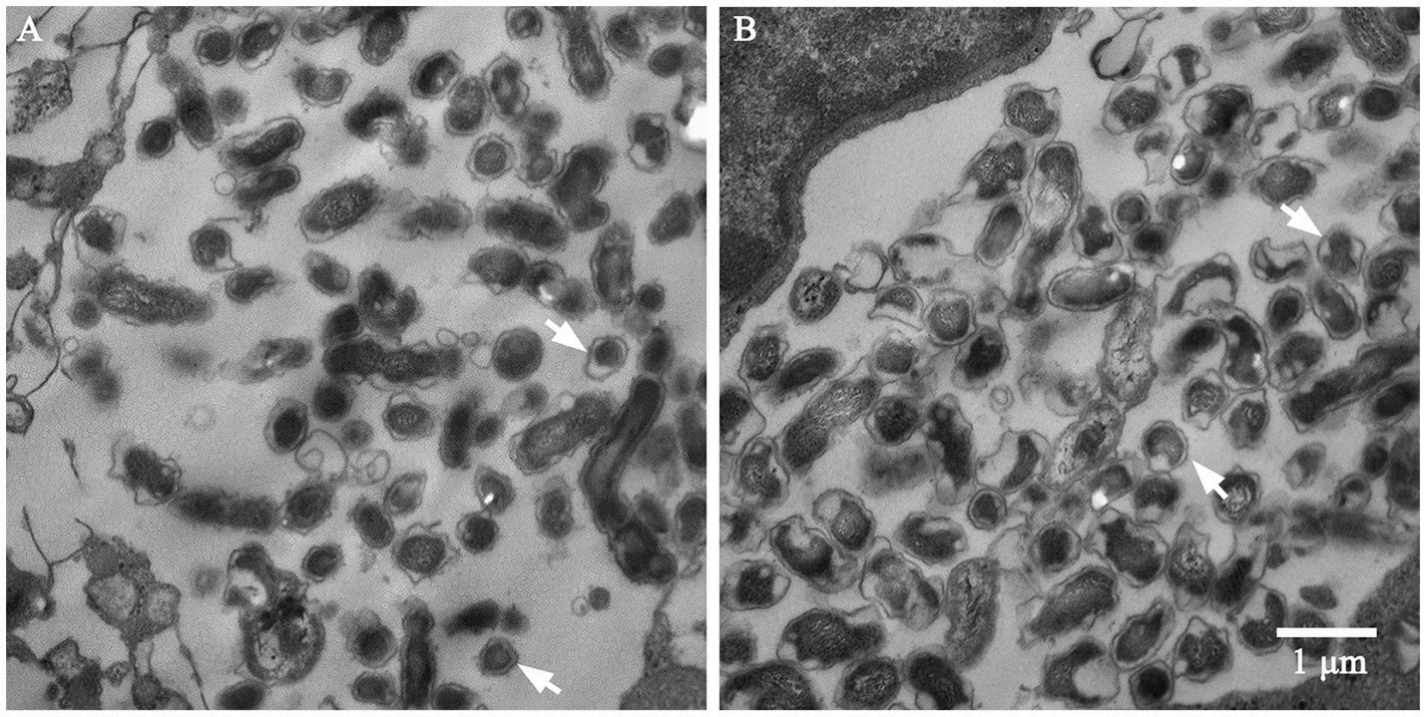
TEM analysis of *C. burnetii* Nine Mile phase II in BGMK cells with **(A)** or without **(B)** inhibitor treatment 7 days post-infection. Bacterial bodies of SCV size can be found in both vacuoles. Arrows indicate SCVs. Scale bar is 1 μm.

In *Chlamydia trachomatis*, LPC-011 can block the transition from replicative reticulate body to infectious elementary body forms (Nguyen et al., 2011). Inhibiting lipid A biosynthesis might also relate to the structural transition of *C. burnetii*, though its two structural forms are both infectious, as indicated by the reduced number of productive vacuoles. Thus, we performed an ultrastructural analysis by transmission electron microscopy (TEM) to assess the developmental transitions (Figure 7). A portion of SCV-sized (0.2–0.5 μm) bodies can be found in vacuoles of inhibitor-treated cells 7 days post-infection. Whether these SCV-sized bodies express protein markers specific to the SCV morphotype is unknown. The TEM data suggests lipid A is not required for the morphological transition of LCV to SCV. Altogether, inhibiting lipid A biosynthesis has moderate effects on survival and growth of wild type phase I and phase II *C. burnetii* strains in BGMK cells.

### Limited propagation of *C. burnetii* in LPC-011-treated THP-1 cells

**Figure 8:**
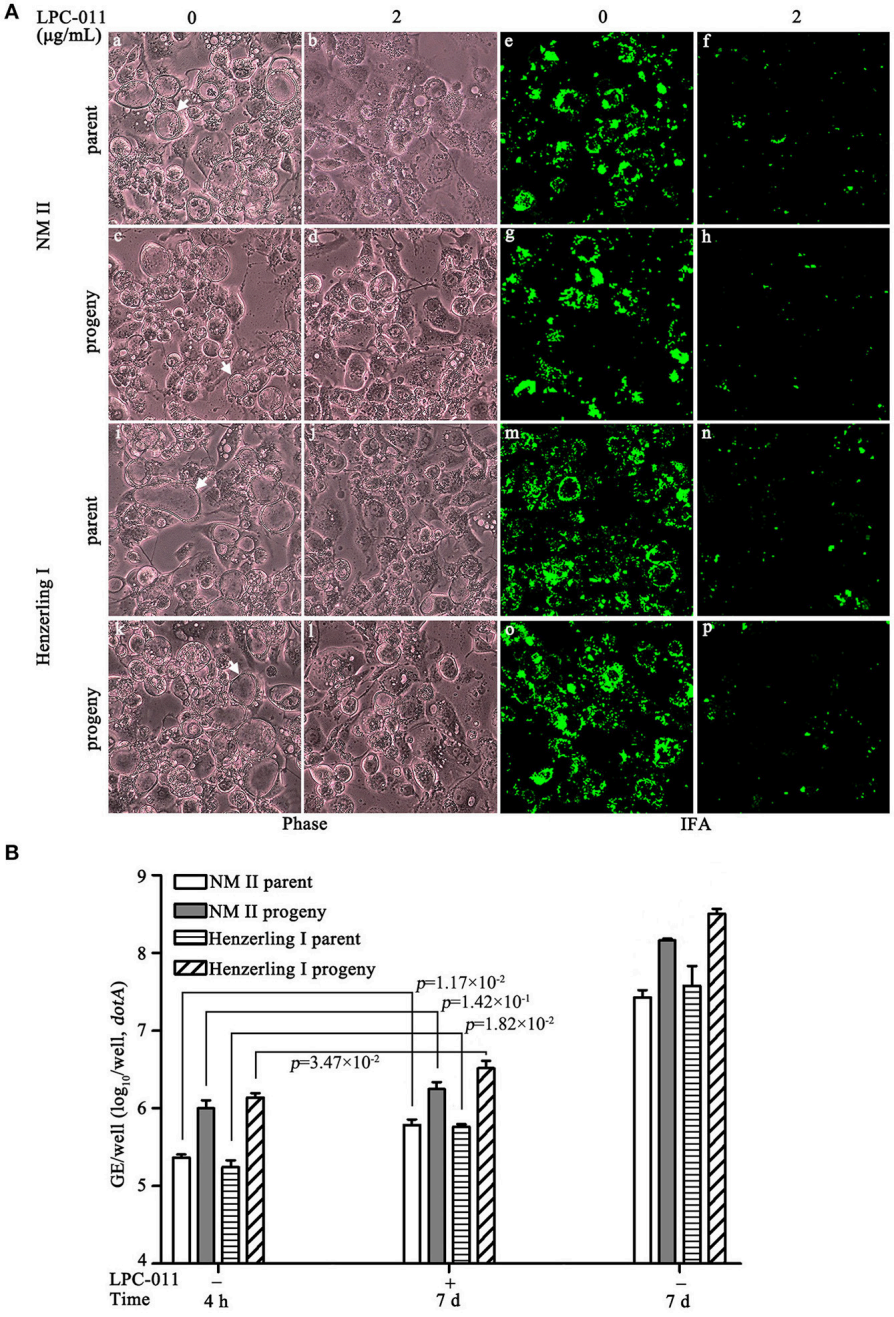
Growth of wild type *C. burnetii* in inhibitor-treated THP-1 cells. **(A)** Four *C. burnetii* strains—Nine Mile phase II, Henzerling phase I and their progenies prepared from inhibitor-treated BGMK cells, were used to infect differentiated THP-1 cells with or without inhibitor treatment. Their growths were assessed by phase microcopy and immunofluorescence. Arrows indicate CCVs. **(B)** Growth yields of the above infections on THP-1 were quantified by qPCR.

The *Cb:Ct kdtA* strain, especially its progeny prepared with inhibitor treatment, has severe growth defects in THP-1. Testing growth of wild type *C. burnetii* in inhibitor-treated macrophages is more relevant for evaluating both the inhibitor's therapeutic use for curing Q fever and the role of lipid A in *C. burnetii* biology. In macrophage-like THP-1 cells with LPC-011 treatment, wild type phase I and phase II *C. burnetii* strains, and their progenies prepared from inhibitor-treated BGMK cultures show similar growth defects (Figure 8). They all appear to form small vacuoles albeit with an occurrence of normal size vacuoles, and all their growth yields increased < 0.5 log (Table S2). Compared to the respective genome copies at 4 h post-infection, the growth yield of progenies of Nine Mile phase II under inhibitor treatment only showed a trend toward enhanced progeny (*p* = 0.1), while growth yields of other three *C. burnetii* preparations reached statistical significance (*p* < 0.05) (Figure 8B, Table S2). All four *C. burnetii* preparations also appear to form a reduced number of vacuoles, but we did not quantify their small vacuoles due to enumerating difficulties. In addition, no mature vacuoles filled with *C. burnetii* particles as in cells without inhibitor-treatment can be observed by phase contrast microscopy. Clearly, lipid A plays a more important role for *C. burnetii* survival and growth in macrophages, which was also suggested by the *Cb:Ct kdtA* strain.

## Discussion

LPS is a highly conserved component in the outer membrane of Gram-negative bacteria. Pioneering work in *E. coli* and *Salmonella* established that lipid A and Kdo components of LPS are essential for outer membrane biogenesis and cell viability. However, with the isolation of LPS-deficient mutants of *Neisseria meningitidis, Moraxella catarrhalis*, and *Acinetobacter baumannii*, it has become clear that the essentiality of Kdo-lipid A of Gram-negative bacteria varies considerably, depending on the species and even on the particular strain background (Bos and Tommassen, 2005; Peng et al., 2005; Moffatt et al., 2010; Zhang et al., 2013). Here we provide evidence that lipid A is nonessential for *C. burnetii* survival and growth *in vitro*, and lipid A has variable roles for bacterial robust growth in different culture systems. Lipid A has significance for optimal replication in macrophages, but appears less significant for replication in non-phagocytic epithelial cells and culture in axenic media.

The ACCM culture techniques greatly promote the development of new methods and tools for *C. burnetii* research (Beare, 2012; Omsland, 2012; Beare and Heinzen, 2014). In this case the ACCM techniques will likely aid the construction of *lpxC* null mutants. *C. burnetii* is an obligate intracellular bacterium in nature. *C. burnetii* genes or factors that are dispensable in axenic culture, could play important roles *in vivo*. For example, the type IV secretion system of *C. burnetii* is found to be required for the development of mature CCV (Beare et al., 2011; Carey et al., 2011). In addition, for unknown reasons, the viability of stationary-phase SCV propagated in ACCM-2 is substantially less than that of host cell-propagated SCVs (Sandoz et al., 2016). Thus we prepared all our strains from BGMK cultures and focused on the role of lipid A in *C. burnetii* growth in various cell lines.

*C. burnetii* preferentially infects mononuclear phagocytes, such as alveolar macrophages (Howe et al., 2010). Phagocytes have a repertoire of antimicrobial or bactericidal mechanisms. In the more relevant macrophage-like THP-1 cells with LpxC inhibitor treatment, *C. burnetii* formed small vacuoles and exhibited limited growth. The role of lipid A for both optimal vacuole formation and bacterial growth of *C. burnetii* in THP-1 cells likely reflects some unique adaptations of the organism to resist the antimicrobial functions of its phagolysosome-like CCV. Our findings highlight the significance of using more relevant cells for investigating the importance of lipid A in intracellular pathogens. In macrophage-targeted *Chlamydia trachomatis* LGV biovar L2 434/Bu, lipid A was found to be required for generating infectious particles, but had no effect on either vacuole development or bacterial replication in nonrelevant epithelial cells (Nguyen et al., 2011). It would be of interest to determine whether lipid A of *C. trachomatis* LGV biovar has similar roles in resisting antimicrobial activities of macrophages.

The CCV is a unique niche for *C. burnetii* replication. The maturation of the CCV is a process orchestrated by type IV secretion system and its effectors (Ghigo et al., 2012; Kohler and Roy, 2015). The variable roles of lipid A for *C. burnetii* growth in different cell types coincide with the distinct CCV growth defects under inhibitor treatment. In Gram-negative bacteria, inhibition of lipid A biosynthesis could compromise some essential functions of the cell envelope, such as affecting the assembly and function of membrane proteins (Zhang et al., 2013). While the growth of most CCVs in epithelial cells is not apparently affected, the reduced number of mature CCVs in epithelial cells and the near absence of mature CCVs in phagocytes may reflect the variable essentiality of secreted effectors that are affected by the loss of lipid A. Future experiments will aim at deleting the *lpxC* gene from phase I and phase II *C. burnetii* and determining the physiological rebalance and respective features of both SCV and LCV upon losing lipid A.

CCVs of phase I and phase II *C. burnetii* strains exhibit distinct late-growth morphological features in epithelial cells. Bacterial particles within phase II Nine Mile CCVs have little intravacuolar movement, while particles within phase I Henzerling CCVs exhibit characteristic Brownian-like movement. This contrasting movement feature may be due to the distinct LPS structures of phase I and phase II particles, as shortened LPS is assumed to increase the hydrophobicity of phase II strains. However, it is unexpected that the distinct movement features of phase I and phase II strains were not affected by inhibiting lipid A biosynthesis. Our data suggest that besides the full length LPS, other surface structures like glycosylated proteins might also contribute to the hydrophilicity of phase I strains. Clearly, further experimentation is needed to determine whether LPS biosynthesis in phase I strain infected cells is effectively depleted by the inhibitors.

Antibiotic susceptibility testing of *C. burnetii* is difficult because this organism is a fastidious and slow-growing intracellular bacterium. A number of cell lines such as P388D1 and J774 (murine macrophage-like cell lines), L929 (a murine fibroblast cell line), and HEL (human embryonic lung cells), have been used to test antibiotic activity against *C. burnetii* (Angelakis and Raoult, 2010). Clearly, our findings also indicate the importance of using more relevant cells for testing antibiotic efficacies against intracellular pathogens. In the case of *C. burnetii*, new clinical therapies for chronic Q fever are needed (Mulye et al., 2017). Our finding of profound inhibition of *C. burnetii* growth in THP-1 cells by LpxC inhibitors encourages additional analysis of the use of these inhibitors as potential therapeutics for the treatment of Q fever.

## Author contributions

TW, YY, SL, ZH, ZS, and YJ: data acquisition, data analysis, data interpretation, revising of the manuscript; XL, AO, and PZ: reagent provision, data analysis, data interpretation, revising of the manuscript; TW and LS: data acquisition, data analysis, data interpretation, writing of the manuscript, revising of the manuscript.

### Conflict of interest statement

The authors declare that the research was conducted in the absence of any commercial or financial relationships that could be construed as a potential conflict of interest.

## Supplementary material

Datasheet S1 Complete sequence of the pMMGKkdtA plasmid.

Figure S1 CCV enumeration in *C. burnetii* (MOI = 1) infected BGMK cells with or without inhibitor treatment. **(A)** Representative images of phase contrast microscopy of *C. burnetii* infected BGMK cells with or without inhibitor treatment. Two *C. burnetii* strains –Nine Mile phase II and Henzerling phase I and their progenies prepared from inhibitor-treated BGMK cells were included. Arrows indicate CCVs. **(B)** Quantitative analysis of CCV numbers formed by different *C. burnetii* strains.

Table S1 Oligonucleotides used in this study.

Table S2 qPCR analyses of *C. burnetii* growth in different culture systems with or without inhibitor treatment.
