# Supplementary material for: Lipid A Has Significance for Optimal Growth of Coxiella burnetii in Macrophage-Like THP-1 Cells and to a Lesser Extent in Axenic Media and Non-phagocytic Cells
Source: Front Cell Infect Microbiol. 2018 Jun 8;8:192. doi: 10.3389/fcimb.2018.00192 (PMC6002510; doi:10.3389/fcimb.2018.00192)
Supplement: Datasheet S1 — Complete sequence of the pMMGKkdtA plasmid. [file Data_Sheet_1.DOCX]

Complete sequence of the pMMGKkdtA plasmid:

CttcaaattcccgttgcacatagcccggcaattcctttccctgctctgccataagcgcagcgaatgccgggtaatactcgtcaacgatctgatagagaagggtttgctcgggtcggtggctctggtaacgaccagtatcccgatcccggctggccgtcctggccgccacatgaggcatgttccgcgtccttgcaatactgtgtttacatacagtctatcgcttagcggaaagttcttttaccctcagccgaaatgcctgccgttgctagacattgccagccagtgcccgtcactcccgtactaactgtcacgaacccctgcaataactgtcacgcccccctgcaataactgtcacgaacccctgcaataactgtcacgcccccaaacctgcaaacccagcaggggcgggggctggcggggtgttggaaaaatccatccatgattatctaagaataatccactaggcgcggttatcagcgcccttgtggggcgctgctgcccttgcccaatatgcccggccagaggccggatagctggtctattcgctgcgctaggctacacaccgccccaccgctgcgcggcagggggaaaggcgggcaaagcccgctaaaccccacaccaaaccccgcagaaatacgctggagcgcttttagccgctttagcggcctttccccctacccgaagggtgggggcgcgtgtgcagccccgcagggcctgtctcggtcgatcattcagcccggctcatccttctggcgtggcggcagaccgaacaaggcgcggtcgtggtcgcgttcaaggtacgcatccattgccgccatgagccgatcctccggccactcgctgctgttcaccttggccaaaatcatggcccccaccagcaccttgcgccttgtttcgttcttgcgctcttgctgctgttcccttgcccgctcccgctgaatttcggcattgattcgcgctcgttgttcttcgagcttggccagccgatccgccgccttgttgctccccttaaccatcttgacaccccattgttaatgtgctgtctcgtaggctatcatggaggcacagcggcggcaatcccgaccctactttgtaggggagggcgcacttaccggtttctcttcgagaaactggcctaacggccacccttcgggcggtgcgctctccgagggccattgcatggagccgaaaagcaaaagcaacagcgaggcagcatggcgatttatcaccttacggcgaaaaccggcagcaggtcgggcggccaatcggccagggccaaggccgactacatccagcgcgaaggcaagtatgcccgcgacatggatgaagtcttgcacgccgaatccgggcacatgccggagttcgtcgagcggcccgccgactactgggatgctgccgacctgtatgaacgcgccaatgggcggctgttcaaggaggtcgaatttgccctgccggtcgagctgaccctcgaccagcagaaggcgctggcgtccgagttcgcccagcacctgaccggtgccgagcgcctgccgtatacgctggccatccatgccggtggcggcgagaacccgcactgccacctgatgatctccgagcggatcaatgacggcatcgagcggcccgccgctcagtggttcaagcggtacaacggcaagaccccggagaagggcggggcacagaagaccgaagcgctcaagcccaaggcatggcttgagcagacccgcgaggcatgggccgaccatgccaaccgggcattagagcgggctggccacgacgcccgcattgaccacagaacacttgaggcgcagggcatcgagcgcctgcccggtgttcacctggggccgaacgtggtggagatggaaggccggggcatccgcaccgaccgggcagacgtggccctgaacatcgacaccgccaacgcccagatcatcgacttacaggaataccgggaggcaatagaccatgaacgcaatcgacagagtgaagaaatccagaggcatcaacgagttagcggagcagatcgaaccgctggcccagagcatggcgacactggccgacgaagcccggcaggtcatgagccagaccaagcaggccagcgaggcgcaggcggcggagtggctgaaagcccagcgccagacaggggcggcatgggtggagctggccaaagagttgcgggaggtagccgccgaggtgagcagcgccgcgcagagcgcccggagcgcgtcgcgggggtggcactggaagctatggctaaccgtgatgctggcttccatgatgcctacggtggtgctgctgatcgcatcgttgctcttgctcgacctgacgccactgacaaccgaggacggctcgatctggctgcgcttggtggcccgatgaagaacgacaggactttgcaggccataggccgacagctcaaggccatgggctgtgagcgcttcgatatcggcgtcagggacgcacccaccggccagatgatgaaccgggaatggtcagccgccgaagtgctccagaacacgccatggctcaagcggatgaatgcccagggcaatgacgtgtatatcaggcccgccgagcaggagcggcatggtctggtgctggtggacgacctcagcgagtttgacctggatgacatgaaagccgagggccgggagcctgccctggtagtggaaaccagcccgaagaactatcaggcatgggtcaaggtggccgacgccgcaggcggtgaacttcgggggcagattgcccggacgctggccagcgagtacgacgccgacccggccagcgccgacagccgccactatggccgcttggcgggcttcaccaaccgcaaggacaagcacaccacccgcgccggttatcagccgtgggtgctgctgcgtgaatccaagggcaagaccgccaccgctggcccggcgctggtgcagcaggctggccagcagatcgagcaggcccagcggcagcaggagaaggcccgcaggctggccagcctcgaactgcccgagcggcagcttagccgccaccggcgcacggcgctggacgagtaccgcagcgagatggccgggctggtcaagcgcttcggtcatgacctcagcaagtgcgactttatcgccgcgcagaagctggccagccggggccgcagtgccgaggaaatcggcaaggccatggccgaggccagcccagcgctggcagagcgcaagcccggccacgaagcggattacatcgagcgcaccgtcagcaaggtcatgggtctgcccagcgtccagcttgcgcgggccgagctggcacgggcaccggcaccccgccagcgaggcatggacaggggcgggccagatttcagcatgtagtgcttgcgttggtactcacgcctgttatactatgagtactcacgcacagaagggggttttatggaatacgaaaaaagcgcttcagggtcggtctacctgatcaaaagtgacaagggctattggttgcccggtggctttggttatacgtcaaacaaggccgaggctggccgcttttcagtcgctgatatggccagccttaaccttgacggctgcaccttgtccttgttccgcgaagacaagcctttcggccccggcaagtttctcggtgactgatatgaaagaccaaaaggacaagcagaccggcgacctgctggccagccctgacgctgtacgccaagcgcgatatgccgagcgcatgaaggccaaagggatgcgtcagcgcaagttctggctgaccgacgacgaatacgaggcgctgcgcgagtgcctggaagaactcagagcggcgcagggcgggggtagtgaccccgccagcgcctaaccaccaactgcctgcaaaggaggcaatcaatggctacccataagcctatcaatattctggaggcgttcgcagcagcgccgccaccgctggactacgttttgcccaacatggtggccggtacggtcggggcgctggtgtcgcccggtggtgccggtaaatccatgctggccctgcaactggccgcacagattgcaggcgggccggatctgctggaggtgggcgaactgcccaccggcccggtgatctacctgcccgccgaagacccgcccaccgccattcatcaccgcctgcacgcccttggggcgcacctcagcgccgaggaacggcaagccgtggctgacggcctgctgatccagccgctgatcggcagcctgcccaacatcatggccccggagtggttcgacggcctcaagcgcgccgccgagggccgccgcctgatggtgctggacacgctgcgccggttccacatcgaggaagaaaacgccagcggccccatggcccaggtcatcggtcgcatggaggccatcgccgccgataccgggtgctctatcgtgttcctgcaccatgccagcaagggcgcggccatgatgggcgcaggcgaccagcagcaggccagccggggcagctcggtactggtcgataacatccgctggcagtcctacctgtcgagcatgaccagcgccgaggccgaggaatggggtgtggacgacgaccagcgccggttcttcgtccgcttcggtgtgagcaaggccaactatggcgcaccgttcgctgatcggtggttcaggcggcatgacgccgtggtgaagcctaagaacaagcacagcctcagccacgtccggcacgacccggcgcactgtctggcccccggcctgttccgtgccctcaagcggggcgagcgcaagcgcagcaagctggacgtgacgtatgactacggcgacggcaagcggatcgagttcagcggcccggagccgctgggcgctgatgatctgcgcatcctgcaagggctggtggccatggctgggcctaatggcctagtgcttggcccggaacccaagaccgaaggcggacggcagctccggctgttcctggaacccaagtgggaggccgtcaccgctgaatgccatgtggtcaaaggtagctatcgggcgctggcaaaggaaatcggggcagaggtcgatagtggtggggcgctcaagcacatacaggactgcatcgagcgcctttggaaggtatccatcatcgcccagaatggccgcaagcggcaggggtttcggctgctgtcggagtacgccagcgacgaggcggacgggcgcctgtacgtggccctgaaccccttgatcgcgcaggccgtcatgggtggcggccagcatgtgcgcatcagcatggacgaggtgcgggcgctggacagcgaaaccgcccgcctgctgcaccagcggctgtgtggctggatcgaccccggcaaaaccggcaaggcttccatagataccttgtgcggctatgtctggccgtcagaggccagtggttcgaccatgcgcaagcgccgcaagcgggtgcgcgaggcgttgccggagctggtcgcgctgggctggacggtaaccgagttcgcggcgggcaagtacgacatcacccggcccaaggcggcaggctgaccccccccactctattgtaaacaagacatttttatcttttatattcaatggcttattttcctgctaattggtaataccatgaaaaataccatgctcagaaaaggcttaacaatattttgaaaaattgcctactgagcgctgccgcacagctccataggccgctttcctggctttgcttccagatgtatgctcttctgctcccgaacCTCGAGTTATTAGATTTTCATGCAAGTAATTTGGCTCTTTAATATTTCCCATGTCTGTTGAAAGCTATTTTCTTCCTGCTTCAAAAAAGATTTCCCCTTTTCAATATAAGCTTGGCGGTTTTTTTCGTTTTGAAGAAGATCTGTAACTACATCTAAAAGAGTTTCTTTGTTCACAGAAAGTCCTGCTTCTTTTTCCCGCAACCTTTCAGCAAGAACAGATTGAGAATAAATATAAGGTCCAAACATAAGAGGCGCCTCTTTTTGAAGAGGCTCTAATAAATTATGCCCTCCTACAGATGGATCGAAAGTTCCTCCTACAAATGCAATATCTGCTGCAGAGTAAATATCTTTTAATACCCCCATAGCGTCCATAATTAAAGAATTATATTGCCGGAAAGAAGCACCTTGACTCCATAATCCAAATAAAATCCCAGCCTTTTCTAGTAGCTTAGCATGCTCCTTTAACTTCTCAAGATGTCTAGGAACCCATAAAATTTTTGTAGAAGAATTGTGGAAATGTGAAACCACTTCAGCCCATACCTCTACATCTTTAGGATGCATAGAACCCAATACAATTAAGCGATCCTGAGAAGATATTTGTAATTTTGCTCTCCAAAAATCTCGGCGATTTGTGGCAAGAGAGGATTCTATAAACGTTTTCATGTTTCCAGTGACATGAATCTTATCCGAGGAAATACCTATCTGCATAAACCGTTGTTTATACAGCTCGTCTTGTAGTATCAATAAATCTAACGGAGCAAAATAATTCCTTCCTAACCGCTTTAAAAAAGAAAAACGCTTACAAGAATGTTCCGAAAGTTTGCCATTAATCAAAAAGGCTTTTGCCCCTAAACGTTTGGATTCTGTCAAAAAATGTAACCAACAGTCTCCTTCTGAAAAGATCACGATATCAGGAGCAAGCTTGCGAACCACGGATTTAATAATACAACTTAAATCCAGAGGTAAAACAAATACAGTAGCGCCAAGAGATTCATACAAACGACGAGCGGTGTGCACACCAGCTTCAGAACACGTTGTAACAACAAACCGCCATTCTGGAAACTCTTCTCTCCATCGATTAAGAAGGGGAGCCAACAGACTGACTTCCCCCACAGAAGCTCCATGAAACCAAACTAAAGGGCCTTCCCCTTTCACGAAAGGTTTCTGTACCCCAAACCGAATTTTCCAAGAGTCAATATATTTCCCATGGAAAAACACTTTGTAAAAAATACGTGGCGCCGAAACAAAAAAAGCGCAAACTAAAAACGCATCATAGAGACGAGATGTTAACCAACGTCTTATCATATGCGCTCTCCTTAACCAACGTCTTATCATATGcgctctcctttcagaaggattaatgtcattatttatttatggggtatggagagggatatttcaaggcgcacaaatatgattccaggctcgttctgcggttccgcctgggtgttcgctgagataggtttttacttcgttatacgaaagcggcgaatcgccaaaaattttttttccaagttcgctgcgaaacgaagccatttcccgaagaagtatgtgccaagtgtttgtaggttttgttaaaattttctaataaggataatagttcattgcacttgtccgtcacaaaggttattttatccacaaaaaatttgcctactaggccctccatttcaggagataagaacgcatcatttatccatttgagtttttggaatggtaaagtttttaaaaaatcatctatctctgaggtttctttagatagaagctttggtagaaaaacattgtccggattaattagagaacctgtttGCTAGCTTACTTGTACAGCCAATGTTGGTACCgattattaattcaaacgggtcaggatttcatgcaggcaaaacctattttagaaattaatccatcgcaccccttaattttacgggtgaaaaatgaatctgataaaacacgctttaatcgttgggccgatttattgcttaatcaagcgctcctagctgaaggagagcagcttaaagaccctgccagttttgtgaaagggctaaatgaattattattagattcctaattacacaaaatagaaagcaaatgcccattaattaaacggattgtttaattaatgggcattttttttttgtaattgaatatccttaattatcggtagaagcttagatttttcttattttaatgattttatgggatttaagtgttgacatttaaaaaagatatcgatattcttcattttatagctttgagaaaagctaagataggcttaaatataggcttattttgattttgttttaactttagttgaaaacgGTTGAAAACGGAGATTTGACATGGTGAGCAAGGGCGAGGAGCTGTTCACCGGGGTGGTGCCCATCCTGGTCGAGCTGGACGGCGACGTAAACGGCCACAAGTTCAGCGTGTCCGGCGAGGGCGAGGGCGATGCCACCTACGGCAAGCTGACCCTGAAGTTCATCTGCACCACCGGCAAGCTGCCCGTGCCCTGGCCCACCCTCGTGACCACCCTGACCTACGGCGTGCAGTGCTTCAGCCGCTACCCCGACCACATGAAGCAGCACGACTTCTTCAAGTCCGCCATGCCCGAAGGCTACGTCCAGGAGCGCACCATCTTCTTCAAGGACGACGGCAACTACAAGACCCGCGCCGAGGTGAAGTTCGAGGGCGACACCCTGGTGAACCGCATCGAGCTGAAGGGCATCGACTTCAAGGAGGACGGCAACATCCTGGGGCACAAGCTGGAGTACAACTACAACAGCCACAACGTCTATATCATGGCCGACAAGCAGAAGAACGGCATCAAGGTGAACTTCAAGATCCGCCACAACATCGAGGACGGCAGCGTGCAGCTCGCCGACCACTACCAGCAGAACACCCCCATCGGCGACGGCCCCGTGCTGCTGCCCGACAACCACTACCTGAGCACCCAGTCCGCCCTGAGCAAAGACCCCAACGAGAAGCGCGATCACATGGTCCTGCTGGAGTTCGTGACCGCCGCCGGGATCACTCTCGGCAATGGACGAGCTGTACAAGTAAaaacaggttctctaattaatccggacaatgtttttctaccaaagcttctatctaaagaaacctcagagatagatgattttttaaaaactttaccattccaaaaactcaaatggataaatgatgcgttcttatctcctgaaatggagggcctagtaggcaaattttttgtggataaaataacctttgtgacggacaagtgcaatgaactattatccttattagaaaattttaacaaaacctacaaacacttggcacatacttcttcgggaaatggcttcgtttcgcagcgaacttggaaaaaaaatttttggcgattcgccgctttcgtataacgaagtaaaaacctatctcagcgaacacccaggcggaaccgcagaacgagcctggaatcatatttgtgcgccttgaaatatccctctccatCTGAAAGGAGAGCGCATATGATTGTCTGAAAGGAGAGCGCATATGATTGAACAAGATGGATTGCACGCAGGTTCTCCGGCCGCTTGGGTGGAGAGGCTATTCGGCTATGACTGGGCACAACAGACAATCGGCTGCTCTGATGCCGCCGTGTTCCGGCTGTCAGCGCAGGGGCGCCCGGTTCTTTTTGTCAAGACCGACCTGTCCGGTGCCCTGAATGAACTGCAGGACGAGGCAGCGCGGCTATCGTGGCTGGCCACGACGGGCGTTCCTTGCGCAGCTGTGCTCGACGTTGTCACTGAAGCGGGAAGGGACTGGCTGCTATTGGGCGAAGTGCCGGGGCAGGATCTCCTGTCATCCCACCTTGCTCCTGCCGAGAAAGTATCCATCATGGCTGATGCAATGCGGCGGCTGCATACGCTTGATCCGGCTACCTGCCCATTCGACCACCAAGCGAAACATCGCATCGAGCGAGCACGTACTCGGATGGAAGCCGGTCTTGTCGATCAGGATGATCTGGACGAAGAGCATCAGGGGCTCGCGCCAGCCGAACTGTTCGCCAGGCTCAAGGCGCGCATGCCCGACGGCGAGGATCTCGTCGTGACCCATGGCGATGCCTGCTTGCCGAATATCATGGTGGAAAATGGCCGCTTTTCTGGATTCATCGACTGTGGCCGGCTGGGTGTGGCGGACCGCTATCAGGACATAGCGTTGGCTACCCGTGATATTGCTGAAGAGCTTGGCGGCGAATGGGCTGACCGCTTCCTCGTGCTTTACGGTATCGCCGCTCCCGATTCGCAGCGCATCGCCTTCTATCGCCTTCTTGACGAGTTCTTCTAATAAGGTAC
