# Supplementary figures and images for: Lipid A Has Significance for Optimal Growth of Coxiella burnetii in Macrophage-Like THP-1 Cells and to a Lesser Extent in Axenic Media and Non-phagocytic Cells
Source: Front Cell Infect Microbiol. 2018 Jun 8;8:192. doi: 10.3389/fcimb.2018.00192 (PMC6002510; doi:10.3389/fcimb.2018.00192)

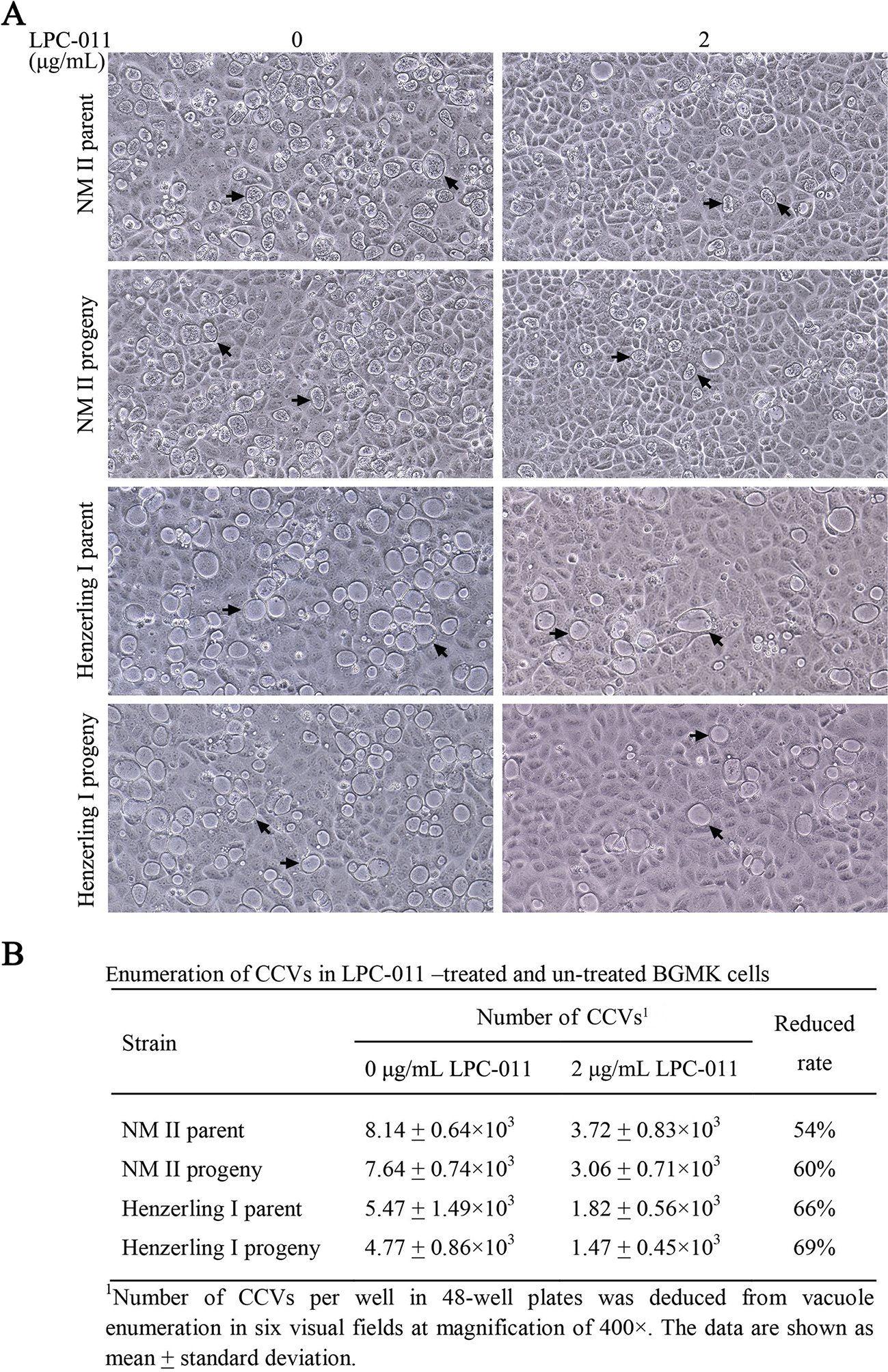

Supplement: Figure S1 — CCV enumeration in C. burnetii (MOI = 1) infected BGMK cells with or without inhibitor treatment. (A) Representative images of phase contrast microscopy of C. burnetii infected BGMK cells with or without inhibitor treatment. Two C. burnetii strains –Nine Mile phase II and Henzerling phase I and their progenies prepared from inhibitor-treated BGMK cells were included. Arrows indicate CCVs. (B) Quantitative analysis of CCV numbers formed by different C. burnetii strains. [file Image_2.TIF]
