# Supplementary material for: Lipid A Has Significance for Optimal Growth of Coxiella burnetii in Macrophage-Like THP-1 Cells and to a Lesser Extent in Axenic Media and Non-phagocytic Cells
Source: Front Cell Infect Microbiol. 2018 Jun 8;8:192. doi: 10.3389/fcimb.2018.00192 (PMC6002510; doi:10.3389/fcimb.2018.00192)
Supplement: Table S1 — Oligonucleotides used in this study. [file Table_1.DOCX]

Table S1 Oligonucleotides used in this study

| Oligo Name | Sequence (5′ to 3′) |
| --- | --- |
| CBlpxCF | GCTGTCATTCAATGACTGAAACC |
| CBlpxCR | TCACGCTGTCATGGCTACCGGGG |
| IBS1/2 | AAAAAAGCTTATAATTATCCTTACGGCACCGCCGCGTGCGCCCAGATAGGGTG |
| EBS1/delta | CAGATTGTACAAATGTGGTGATAACAGATAAGTCCGCCGCTCTAACTTACCTTTCTTTGT |
| EBS2 | TGAACGCAAGTTTCTAATTTCGATTTGCCGTCGATAGAGGAAAGTGTCT |
| EBS/Universal  P311-KpnI-F | CGAAATTAGAAACTTGCGTTCAGTAAAC |
|  | CCAATGTTGGTACCGATTATTAATTCAAACG |
| P311-R | CTCGCCCTTGCTCACCATGTCAAATCTCCGTTTTCAAC |
| P1169-F | ATGGACGAGCTGTACAAGTAAAAACAGGTTCTCTAATTAATC |
| P1169-R | CAATCATATGCGCTCTCCTTTCAG |
| Kan-F | TCTGAAAGGAGAGCGCATATGATTGAACAAGATGGAT |
| Kan-XhoI-R | TTCCTACCTCGAGTTAGAAGAACTCGTCAAGAAG |
| eGFP-F | GTTGAAAACGGAGATTTGACATGGTGAGCAAGGGCGAG |
| eGFP-R | GATTAATTAGAGAACCTGTTTTTACTTGTACAGCTCGTCCAT |
| p207-XhoI-F | TTCAACTCTCGAGAGTTATTGTCTTCAAATTCCCGT |
| p207-KpnI-R | TAGCTGTGGTACCGTGCGGCAGCGCTCAGTAG |
| P1169-NheI-F | CTGTACAAGTAAGCTAGCAAACAGGTTCTCTAATTAATC |
| P1169-kdtA-R | TAACCAACGTCTTATCATATGCGCTCTCCTTTCAG |
| kdtA-NheI-F | AGGAGAGCGCATATGATAAGACGTTGGTTAAC |
| kdtA-XhoI-R | TCTACGACAGATCTCGAGTTATTAGATTTTCATGCAAGTAAT |
| Kan-KpnI-R | CAGCTATTGGTACCTTATTAGAAGAACTCGTCAAGAAG |
